# Advancing transmural remission as a treatment target in Crohn's disease: The future of tight‐control strategy?

**DOI:** 10.1002/ueg2.12495

**Published:** 2023-11-16

**Authors:** Ferdinando D'Amico, Sara Massironi, Mariangela Allocca, Silvio Danese

**Affiliations:** ^1^ Division of Gastroenterology and Endoscopy IRCCS Ospedale San Raffaele Milan Italy; ^2^ Vita‐Salute San Raffaele University Milan Italy; ^3^ Department of Biomedical Sciences Humanitas University Milan Italy; ^4^ Division of Gastroenterology Fondazione IRCCS San Gerardo dei Tintori Monza Italy; ^5^ School of Medicine University of Milano‐Bicocca Monza Italy

**Keywords:** Crohn's disease, inflammatory bowel disease, intestinal ultrasound, tight control, transmural remission

Crohn's disease (CD), a chronic inflammatory bowel disease (IBD), is an ongoing clinical challenge. Tight disease monitoring is increasingly recognized as a key strategy to achieve optimal disease control.^1^ In addition, therapeutic goals in CD have evolved greatly in recent decades to include not only clinical remission but also endoscopic mucosal healing, and more recently even transmural healing (TH).^2^, ^3^ Indeed, considering that transmural damage constitutes a hallmark of CD, it might be more suitable to target TH as assessed through intestinal ultrasonography (IUS) or magnetic resonance enterography (MRE) rather than mere mucosal healing. Of note, TH is emerging as a promising benchmark associated with improved patient outcomes,^2^ including reduced hospitalizations, surgeries, steroid dependency, and treatment escalation.^4^, ^5^, ^6^ Unfortunately, a commonly validated definition of TH is not yet available. To overcome this limitation, an expert consensus on behalf of the International Bowel Ultrasound [IBUS] Group proposed a definition of TH including bowel wall thickness ≤3 mm and the absence of vascular signals on Doppler.^7^ Achieving transmural remission has proven challenging, with no more than 50% of CD patients reaching this milestone, even after advanced therapies,^8^, ^9^ and less than 30% in real‐life studies. A recent study by Fernandes et al.^10^ takes a significant step in elucidating the potential of a tight‐control strategy to optimize CD management to achieve TH. The study underscores that a "tight control" strategy, guided by fecal calprotectin (FC) monitoring and reactive treatment escalation, significantly enhances the rates of transmural remission. The robustness of the study is bolstered by the concurrent use of various investigative modalities, including colonoscopy, MRE, and FC. Patients who experienced an augmented rate of treatment escalation following abnormal FC results were significantly more likely to achieve TH. This implies that adopting a proactive approach to monitoring and adapting treatment in response to biomarker fluctuations may lead to improved clinical outcomes. Notably, the study highlights the importance of early intervention. Patients with early‐stage CD, defined as a disease duration of less than 18 months without prior treatment with immunomodulators and biologics, benefited most from this tight‐control strategy.^11^ Implementing a tight‐control strategy necessitates frequent assessments of FC and other imaging investigations, possibly incurring additional costs. However, while this approach may entail elevated monitoring and treatment costs in the short term, it has the potential to yield substantial cost savings by preventing disease progression and complications in the long run.^12^ Indeed, these upfront costs should be juxtaposed with the potential benefits. The primary benefit of the tight‐control strategy lies in its capacity to promptly identify disease flares and inflammation. Timely intervention enables clinicians to arrest disease progression and mitigate the necessity for more intensive treatments, hospitalizations, and surgeries. The economic benefits associated with averting these severe consequences can be substantial. Moreover, when disease activity is closely monitored, treatment adjustments can be tailored to the patient's immediate needs curtailing the unnecessary use of medications and healthcare resources. Beyond cost considerations, patient‐centered outcomes such as improved quality of life, reduced disease burden, and increased productivity should be included in the overarching societal cost–benefit assessment. While considering the advantages of a tight‐control strategy based on TH, it is imperative to acknowledge its limitations. Various tools allow the evaluation of TH including MRE, IUS, and computed tomography (CT) scans. Comparative studies showed that IUS and MRE have comparable accuracy while avoiding the radiation risk associated with CT scans.^13^, ^14^ However, there is no evidence comparing the outcomes of patients monitored closely using different tools. Importantly, IUS could be the most appropriate method for this approach. IUS stands out as a patient‐centric, user‐friendly, noninvasive, real‐time tool ideally suited for clinical decision‐making in IBD practice.^15^ IUS not only allows the assessment of disease activity and response to treatment but also predicts long‐term endoscopic activity of disease.^16^ Furthermore, its diagnostic accuracy approaches that of more elaborate conventional radiological examinations like MRE.^17^, ^18^ It is worth noting that other studies have already demonstrated the efficacy of clinical decision‐making involving IUS findings in reducing inflammation in patients with IBD,^19^ and IUS is increasingly finding application in CD management,^20^, ^21^, ^22^ underscoring its potential utility as a pivotal tool in striving for transmural remission. Inflammatory bowel disease gastroenterologists should be encouraged to consider transmural remission as a viable treatment goal for CD management. A close monitoring strategy including tight monitoring and prompt treatment adjustment could lead to more favorable long‐term outcomes. Nevertheless, discrepancies exist between clinical, endoscopic, and transmural activity in patients with CD.^23^ For this reason, as also hypothesized in ulcerative colitis, the achievement of an increasingly deep remission that simultaneously includes clinical, endoscopic, and transmural disease activity could be associated with improved disease control and patients' quality of life.^24^

## CONFLICT OF INTEREST STATEMENT

The authors have no conflicts of interest to declare.

## Data Availability

Data sharing is not applicable to this article as no new data were created or analyzed in this study.

## References

[1] Colombel JF , Panaccione R , Bossuyt P , Lukas M , Baert F , Vaňásek T , et al. Effect of tight control management on Crohn's disease (CALM): a multicentre, randomised, controlled phase 3 trial. Lancet. 2017;390(10114):2779–2789. 10.1016/s0140-6736(17)32641-7 29096949

[2] Neurath MF , Vieth M . Different levels of healing in inflammatory bowel diseases: mucosal, histological, transmural, barrier and complete healing. Gut. 2023;72(11):2164–2183. 10.1136/gutjnl-2023-329964 37640443

[3] Turner D , Ricciuto A , Lewis A , D’Amico F , Dhaliwal J , Griffiths AM , et al. STRIDE‐II: an update on the selecting therapeutic targets in inflammatory bowel disease (STRIDE) initiative of the international organization for the study of IBD (IOIBD): determining therapeutic goals for treat‐to‐target strategies in IBD. Gastroenterology. 2021;160(5):1570–1583. 10.1053/j.gastro.2020.12.031 33359090

[4] Lafeuille P , Hordonneau C , Vignette J , Blayac L , Dapoigny M , Reymond M , et al. Transmural healing and MRI healing are associated with lower risk of bowel damage progression than endoscopic mucosal healing in Crohn's disease. Aliment Pharmacol Ther. 2021;53(5):577–586. 10.1111/apt.16232 33368525

[5] Messadeg L , Hordonneau C , Bouguen G , Goutorbe F , Reimund JM , Goutte M , et al. Early transmural response assessed using magnetic resonance imaging could predict sustained clinical remission and prevent bowel damage in patients with Crohn's disease treated with anti‐tumour necrosis factor therapy. J Crohns Colitis. 2020;14(11):1524–1534. 10.1093/ecco-jcc/jjaa098 32533769

[6] Fernandes SR , Serrazina J , Botto IA , Leal T , Guimarães A , Garcia JL , et al. Transmural remission improves clinical outcomes up to 5 years in Crohn's disease. United Eur Gastroenterol J. 2023;11(1):51–59. 10.1002/ueg2.12356 PMC9892415 36575615

[7] Ilvemark J , Hansen T , Goodsall TM , Seidelin JB , Al‐ Farhan H , Allocca M , et al. Defining transabdominal intestinal Ultrasound treatment response and remission in inflammatory bowel disease: systematic review and expert consensus statement. J Crohns Colitis. 2022;16(4):554–580. 10.1093/ecco-jcc/jjab173 34614172 PMC9089416

[8] Kucharzik T , Wilkens R , D'Agostino MA , Maconi G , Le Bars M , Lahaye M , et al. Early Ultrasound response and progressive transmural remission after treatment with ustekinumab in Crohn's disease. Clin Gastroenterol Hepatol. 2023;21(1):153–163.e12. 10.1016/j.cgh.2022.05.055 35842121

[9] Zorzi F , Rubin DT , Cleveland NK , Monteleone G , Calabrese E . Ultrasonographic transmural healing in Crohn's disease. Am J Gastroenterol. 2023;118(6):961–969. 10.14309/ajg.0000000000002265 36988302

[10] Fernandes S . In: Bernardo S , editor. United European Gastro Journal. Wiley; 2023.

[11] Colombel JF , D'Haens G , Lee WJ , Petersson J , Panaccione R . Outcomes and strategies to support a treat‐to‐target approach in inflammatory bowel disease: a systematic review. J Crohns Colitis. 2020;14(2):254–266. 10.1093/ecco-jcc/jjz131 31403666 PMC7008150

[12] Lakatos PL , Kaplan GG , Bressler B , Khanna R , Targownik L , Jones J , et al. Cost‐effectiveness of tight control for Crohn's disease with adalimumab‐based treatment: economic evaluation of the CALM trial from a Canadian perspective. J Can Assoc Gastroenterol. 2022;5(4):169–176. 10.1093/jcag/gwac001 35919766 PMC9340647

[13] Allocca M , Fiorino G , Bonifacio C , Furfaro F , Gilardi D , Argollo M , et al. Comparative accuracy of bowel Ultrasound versus magnetic resonance enterography in combination with colonoscopy in assessing Crohn's disease and guiding clinical decision‐making. J Crohns Colitis. 2018;12(11):1280–1287. 10.1093/ecco-jcc/jjy093 29982361

[14] Lee DI , You MW , Park SH , Seo M . Comparison of diagnostic performance of ultrasonography and magnetic resonance enterography in the assessment of active bowel lesions in patients with Crohn's disease: a systematic review and meta‐analysis. Diagnostics. 2022;12(8):2008. 10.3390/diagnostics12082008 36010359 PMC9407121

[15] Dolinger MT , Kayal M . Intestinal Ultrasound is the ideal patient‐centric, point‐of‐care tool for clinical decision making in the inflammatory bowel disease practice. In: Crohns colitis 360. England; 2023. p. otad029. 10.1093/crocol/otad029 PMC10246577 37292104

[16] Allocca M , Dell’Avalle C , Furfaro F , Zilli A , Radice S , D’Amico F , et al. P330 Ultrasound remission after biologic induction predicts long‐term endoscopic remission in Crohn’s disease. J Crohn's Colitis. 2023;17(Supplment_1):i468. 10.1093/ecco-jcc/jjac190.0460

[17] Rimola J , Beek K , Ordás I , Gecse K , Cuatrecasas M , Stoker J . Contemporary imaging assessment of strictures and fibrosis in Crohn disease, with focus on quantitative biomarkers: from the AJR special series on imaging of fibrosis. AJR Am J Roentgenol. 2023. 10.2214/ajr.23.29693 37530400

[18] Nancey S , Fumery M , Faure M , Boschetti G , Gay C , Milot L , et al. Use of imaging modalities for decision‐making in inflammatory bowel disease. Therap Adv Gastroenterol. 2023;16:17562848231151293. 10.1177/17562848231151293 PMC9912556 36777362

[19] Saleh A , Abraham BP . Utility of intestinal Ultrasound in clinical decision‐making for inflammatory bowel disease. Crohns Colitis 360. 2023;5(3):otad027. 10.1093/crocol/otad027 37292105 PMC10246583

[20] Abraham BP , Reddy D , Saleh A . Integrating intestinal Ultrasound into an inflammatory bowel disease practice: how to get started. Crohns Colitis 360. 2023;5(3):otad043. 10.1093/crocol/otad043 37719309 PMC10500970

[21] Lin WC , Chang CW , Chen MJ , Wang HY . Intestinal Ultrasound in inflammatory bowel disease: a novel and increasingly important tool. J Med Ultrasound. 2023;31(2):86–91. 10.4103/jmu.jmu_84_22 37576427 PMC10413392

[22] Allocca M , Kucharzik T , Rubin DT . Intestinal Ultrasound in the assessment and management of inflammatory bowel disease: is it ready for standard practice? Gastroenterology. 2023;164(6):851–855. 10.1053/j.gastro.2023.01.021 36708790

[23] Onali S , Calabrese E , Petruzziello C , Zorzi F , Sica G , Lolli E , et al. Endoscopic vs ultrasonographic findings related to Crohn's disease recurrence: a prospective longitudinal study at 3 years. J Crohns Colitis. 2010;4:319–328. 10.1016/j.crohns.2009.12.010 21122521

[24] Colombel JF . Disease clearance in inflammatory bowel disease. Gastroenterol Hepatol. 2021;17:233–235. PMC8667380 34924892

